# In vitro interactions of crocin with fluconazole against *Candida* isolates

**DOI:** 10.18502/cmm.4.4.383

**Published:** 2018-12

**Authors:** Narges Aslani, Mohammad Taghi Hedayati, Mojtaba Nabili, Abdolali Faramarzi, Farzaneh Sadeghi, Maryam Moazeni

**Affiliations:** 1 Infectious and Tropical Diseases Research Center, Tabriz University of Medical Sciences, Tabriz, Iran; 2 Invasive Fungi Research Centre, School of Medicine, Mazandaran University of Medical Sciences, Sari, Iran; 3 Department of Medical Mycology and Parasitology, School of Medicine, Mazandaran University of Medical Sciences, Sari, Iran; 4 Department of Medical Sciences, Islamic Azad University, Sari Branch, Sari, Iran; 5 Neurocognitive Research Center, Faculty of Medicine, Mashhad University of Medical Sciences, Mashhad, Iran; 6 Student Research Committee, Mazandaran University of Medical Sciences, Sari, Iran

**Keywords:** Candida, Combination, Crocin, Fluconazole

## Abstract

**Background and Purpose:**

The incidence of invasive fungal infections has been increased in recent years. The growing use of azole drugs for prophylactic and therapeutic purposes has resulted in the gradual emergence of azole-resistant species. Accordingly, the introduction of a new strategy to improve the management of *Candida* infections is an urgent need. Regarding this, the present study was performed to evaluate the antifungal activities of crocin (Cro) alone and in combination with fluconazole.

**Materials and Methods:**

**This study was conducted on 50 **clinical isolates of four different *Candida *species. The identity of the isolates was confirmed using the internal transcribed spacer identification system. The interactions of Cro with fluconazole were investigated using a microdilution checkerboard method based on the Clinical and Laboratory Standards Institute reference technique with 96-well microtiter plates. Furthermore, the assessment of the interaction of drug combinations was accomplished using the fractional inhibitory concentration index (FICI) based on the Loewe additivity theory.

**Results:**

According to the results, Cro alone showed a relatively high MIC50 value (1 g/ml) against *Candida* species. Our results demonstrated indifferent interactions between Cro and fluconazole with a FICI range of 0.5-4 against *Candida* strains.

**Conclusion:**

The high MIC value for Cro against *Candida* species indicated its failure to show appropriate antifungal activity against this species. The MIC of this agent was not significantly reduced even by the addition of fluconazole. Therefore, other mechanisms which are not related to the mechanism of azole drugs are involved at high concentration of Cro.

## Introduction

Candidiasis is a serious life-threatening infection and will undoubtedly continue to grow in parallel with the significantly increasing number of patients receiving medical care, especially those with immunodeficiency [1-3]. There are a number of antifungal agents available for the management of candidiasis, including azoles, polyenes, and echinocandins; however, these medications are still limited [4-8]. The toxic effects of amphotericin B and emergence of drug resistant isolates of *Candida* species, mostly azole- and echinocandin-resistant species, have recently become a serious clinical challenge [9-12]. 

Azoles have been used for many years as the first-line therapy for the treatment of *Candida* infections, antifungal prophylaxis, and empirical or pre-emptive treatment given their good safety profile and high therapeutic index. Among the azole drugs, fluconazole is the most widely used agent for systemic candidiasis due to its high solubility, low toxicity, and wide tissue distribution [13]. 

However, it seems that the increasing clinical use of fluconazole for prophylactic and therapeutic purposes has resulted in the gradual emergence of azole-resistant species [1]. Accordingly, it is required to introduce a new antifungal formulation [14, 15] or investigate the combination of two or more antifungal drugs with potent activities to improve the management of *Candida* infections [16]. 

Synergistic properties of combination therapy contribute to the enhancement of antifungal activities. Combination of natural antimicrobials with synthetic chemical therapeutic agents (e.g., fluconazole as the first-line therapy against candidiasis) may help develop new antifungal drugs. Currently, it is assumed that natural products have potent synergistic activities against fungi when combined with an antifungal agent. 

Saffron flower (*Crocus sativus*) is currently used with a wide range of applications, such as a source of food additive, colorant, and component of traditional medicines. Studies have shown that Cro isolated from *C. sativus* has numerous beneficial properties, including remarkable antibacterial, antioxidant, and antitumoural effects; moreover, it may have cardioprotective effects [17]. However, to the best of our knowledge, there is limited information regarding the antifungal activity of this plant. With this background in mind, the present study was conducted to evaluate the synergistic antifungal effect of Cro combined with fluconazole on *Candida* infections.

## Materials and Methods

***Isolates***

This study was conducted on 50 *Candida *isolates from four different types of fluconazole-susceptible (n=40) and fluconazole-resistant (n=10) species. The isolates were obtained from the Reference Culture Collection of Invasive Fungi Research Center (IFRC) in Sari, Iran (Table 1). Although the isolates had been previously identified, their identities were confirmed through sequencing the internal transcribed spacer ribosomal DNA region. Moreover, the susceptibility pattern of the isolates to flocunazole had been registered at the IFRC database previously. Frozen stocks of isolates were stored at -80ºC in a culture medium supplemented with 40% (vol/vol) glycerol, and then subcultured twice at 35ºC before each experiment. 

***In vitro susceptibility testing***

Fluconazole (Pfizer, Groton, CT, USA) was obtained from the respective manufacturers in form of powder and used for the preparation of the Clinical and Laboratory Standards Institute (CLSI) microdilution trays. The Cro (98% purity) was obtained from Mashhad University of Medical Sciences, Mashhad, Iran. It was stored at a concentration of 2 mg/ml in dimethyl sulfoxide (DMSO). Furthermore, fluconazole was prepared at a final concentration of 128-0.125 µg/ml. 

Antifungal susceptibility testing of the yeast strains to fluconazole and Cro was performed according to the CLSI guidelines documents M27-A3 and M27-S4 [18, 19]. The RPMI 1640 medium with glutamine without bicarbonate (Sigma) buffered at a pH of 7 with 0.165 mol/l 3- N-morpholinepropanesulfonic acid (MOPS; Sigma) was used. Drug-free and yeast-free controls were also included in the study. The microtiter plates were incubated at 35°C and read visually after 24 h. The quality control strains, namely *C. krusei* ATCC 6258 and *C. parapsilosis* ATCC 22019, were used in each susceptibility test to ensure quality control.

***Checkerboard microdilution assay***

Interactions of Cro with fluconazole were investigated using a microdilution checkerboard technique in a 96-well microtitre plate. Drug dilutions were prepared to obtain four times the final concentration. Concentrations of fluconazole and Cro had the ranges of 8-0.016 µg/ml and 1-0.063 mg/ml, respectively. For the two-dimensional microplate preparation (i.e., fluconazole plus Cro), 50 µl of each concentration of Cro was added to columns 1-11. Subsequently, 50 µl of fluconazole was added to rows A to H, respectively. 

The wells of column 11 and row H entailed 50 µl of RPMI medium containing 1% of the solvent. Row H and column 11 contained the fluconazole and Cro alone, respectively. Column 12 was the drug-free well that served as the growth control. The model-fractional inhibitory concentration index (FICI) method was applied to interpret the results. The FICI was calculated by the following equation: 

FICI=FIC A+FIC B

where, FIC A is the ratio of the MIC of the combination to the MIC of drug A alone, and FIC B is the ratio of MIC of the combination to the MIC of drug B alone. The interaction was defined as synergistic, indifferent, and antagonistic if the FICIs were ≤ 0.5, > 0.5 to ≤ 4.0, and > 4, respectively [20].

***Time-killing assay***

*Candida albicans* isolates were prepared with an initial inoculum of 10^5^ cells/ml. The Cro concentrations were 2 and 1 mg/ml, and fluconazole concentrations were 4, 8, and 16 µg/ml. The DMSO comprised more than 1% of the total test volume. At predetermined time points (i.e., 0, 2, 4, and 24 h) after incubation with agitation at 35ºC, 100 ml of aliquot was removed from every solution and serially diluted by 10-fold in sterile water. In the next stage, 100 ml aliquot from each dilution was spread on the sabouraud dextrose agar plate. 

Colony counts were determined after incubation at 35ºC for 48 h. Fungicidal activity was defined as ≥ 3 log 10 reduction from the initial inoculum. Synergism and antagonism were defined as a respective decrease or increase of ≥ 2 log10 CFU/ml in antifungal activity produced by the combined preparation, compared with that of the more active agent alone [21].

## Results

***In vitro susceptibility testing***

The MIC values of Cro tested alone or in combination with fluconazole against 50 clinical *Candida *isolates obtained from four different species are shown in Table 1. The MICs for Cro against all tested strains were 1 mg/ml. According to the analysis of FICI method, the combination of Cro with fluconazole exhibited indifferent activity against all tested strains (>0.5-≤4.0).

**Table 1:** Interactions between fluconazole and crocin against 50 clinical isolates of *Candida* from four different species

**Isolates**	**MIC**			
	**Fluconazole ** * ** (µg/ml)**	**Cro** ** ** (mg/ml)**	**Fluconazole /Cro**	**FICI** ¥ **/INT** £
*C. alb*1	1.0	1.0	1.0/0.25	1.25/Ind €
*C.alb*2	0.5	1.0	0.5/0.25	1.25/Ind
*C. alb*3	2	1.0	1.0/0.25	0.75/Ind
*C. alb*4	0.5	1.0	0.5/0.25	2.25/Ind
*C. alb*5	1.0	1.0	0.5/0.125	0.625/ Ind
*C. alb*6	0.5	1.0	0.5/0.125	1.125/ Ind
*C. alb*7	1.0	1.0	0.5/0.125	0.625/ Ind
*C. alb*8	2.0	1.0	2.0/0.025	1.25/ Ind
*C. alb*9	1.0	1.0	1.0/0.25	1.25/ Ind
*C. alb*10	0.5	1.0	0.5/0.25	1.25/ Ind
*C. alb*R1	8.0	1.0	8.0/0.25	1.25/ Ind
*C. alb*R2	8.0	1.0	8.0/0.25	1.25/ Ind
*C. alb*R3	8.0	1.0	8.0/0.125	1.125/ Ind
*C. alb*R4	8.0	1.0	8.0/0.5	3.0/ Ind
*C. alb*R 5	8.0	1.0	8.0/0.25	1.25/ Ind
*C.gla*1	0.25	1.0	0.25/0.25	1.25/ Ind
*C.gla*2	0.25	1.0	0.125/0.5	1.0/ Ind
*C.gla*3	2.0	1.0	2.0/0.5	1.5/ Ind
*C.gla*4	2.0	1.0	2.0/0.5	1.5/ Ind
*C.gla*5	4.0	1.0	4.0/0.125	1.125/ Ind
*C.gla* 6	2.0	1.0	2.0/0.5	1.5/ Ind
*C.gla*7	4.0	1.0	4.0/0.25	1.25/ Ind
*C.gla*8	2.0	1.0	2.0/0.5	1.5/ Ind
*C.gla*9	4.0	1.0	4.0/1.0	2.0/ Ind
*C.gla* 10	4.0	1.0	4.0/0.125	1.125/ Ind
*C.gla*R1	64.0	1.0	16.0/1.0	1.25/ Ind
*C.gla*R 2	64.0	1.0	16.0/0.5	0.75/ Ind
*C.gla*R 3	64.0	1.0	16.0/1.0	1.25/ Ind
*C.gla*R 4	64.0	1.0	16.0/1.0	1.25/ Ind
*C.gla*R 5	64.0	1.0	16.0/1.0	1.25/ Ind
*C. para*1	0.125	1.0	0.125/0.125	1.125/ Ind
*C. para*2	0.25	1.0	0.125/0.5	1.0/ Ind
*C. para*3	0.5	1.0	0.25/0.5	1.0/ Ind
*C. para*4	0.125	1.0	0.125/0.5	1.5/ Ind
*C. para*5	0.125	1.0	0.125/0.5	1.5/ Ind
*C. para*6	0.125	1.0	0.125/0.125	1.125/ Ind
*C. para*7	0.25	1.0	0.125/0.5	1.0/ Ind
*C. para*8	0.25	1.0	0.25/0.25	1.25/ Ind
*C. para*9	0.5	1.0	0.5.0/0.25	1.25/ Ind
*C. para*10	0.25	1.0	0.25/0.25	1.25/ Ind
*C. dub*1	0.125	1.0	0.125/0.5	1.5/ Ind
*C. dub*2	0.25	1.0	0.25/1.0	2.0/ Ind
*C. dub*3	0.25	1.0	0.125/0.5	1.0/ Ind
*C. dub*4	0.25	1.0	0.25/0.25	1.25/ Ind
*C. dub*5	0.25	1.0	0.125/0.5	1.0/ Ind
*C. dub*6	0.125	1.0	0.125/0.5	1.5/ Ind
*C. dub*7	0.25	1.0	0.25/0.25	1.25/ Ind
*C. dub*8	0.25	1.0	0.125/0.5	1/ Ind
*C. dub*9	0.25	1.0	0.25/0.25	1.25/ Ind
*C. dub*10	0.125	1.0	0.125/0.5	1.5/ Ind

* Fluconazole,

** Cro: Crocin,

¥ FICI: fractional inhibitory concentration index,

£ INT: Interaction,

€ Ind: indifferent interaction

***Investigation of fungicidal effect by time-killing test***

**Figure 1:**
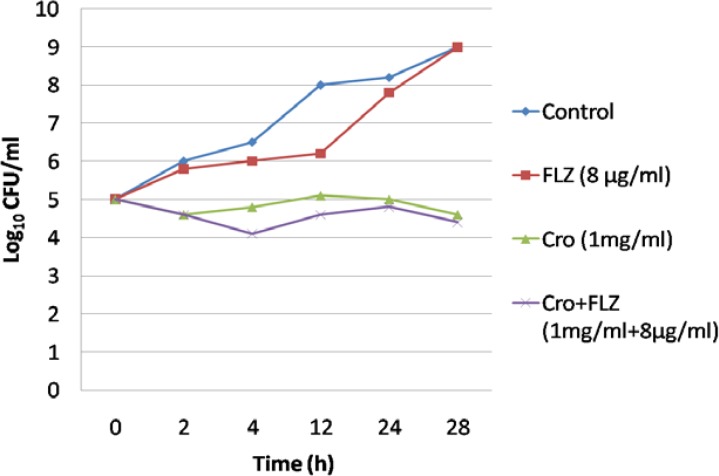
Interaction of crocin (1 µg/ml) combined with fluconazole at three concentrations by time-killing test (After 24 h of incubation, the isolate behaved as control sample when exposed to fluconazole. Although the growth rate decreased when it was treated with crocin, no synergistic effect was observed as the growth rate did not decrease since synergism and antagonism are defined as a respective decrease or increase of ≥ 2 log10 CFU/ml in antifungal activity.)

Cells were inoculated onto a potato dextrose agar plate to count the colony forming unit for determining synergistic fungicidal effect. The interaction of Cro with fluconazole at a concentration of 1 mg/ml was confirmed by the time-killing test (Figure 1). However, no appreciable antifungal activity was observed, and a complete cell-killing was not achieved. 

## Discussion

Considering the large population of high-risk individuals, the incidence of severe candidiasis has undergone a dramatic increase [2, 3]. Prolonged use of fluconazole as the first-line therapy, for the prophylaxis and treatment of this infection has contributed to the development of drug resistance in *Candida *isolates. Accordingly, the combination of two or more antifungal agents may be a good alternative and feasible policy to solve this problem. 

A large number of natural products originated from plants are reported to possess potent antifungal properties in recent years, such as terpene derivatives, flavans, nucleosides, peptides, alkaloids, saponins, and sterols [22]. Many reports have indicated the antimicrobial activities of saffron flower extract as a natural product as a result of its safranal and Cro compounds [23]. 

A study involved the exploration of the anti-*Candida* effects of two bioactive compounds obtained from *Crocus sativus* *stigmas*, namely Cro 1 and safranal. In the mentioned study, some semisynthetic derivatives of safranal led to promising biological results in terms of MIC/minimum fungicidal concentration values, synergism, and reduction in the germ tube formation [24]. 

In another study, the ethyl acetate extract of Cro was found to inhibit the growth of such microorganisms as *Escherichia coli, Pseudomonas aeruginosa, Staphylococcus aureus, Yersinia enterocolitica, *and *C. albicans* [25, 26]. Liu et al. investigated glabridin as a natural product that is an isoflavan obtained from *Glycyrrhiza glabra* root. They reported that glabridin had a weak antifungal activity against different fungi, such as *Candida* species and *Cryptococcus* *neoformans* [27-29]. To the best of our knowledge, there is no investigation on the combination of Cro and fluconazole against fungi. 

## Conclusion

In contrast to other publications, a high MIC value was obtained against *Candida* species when using Cro alone. To reduce this concentration, fluconazole was applied in combination with Cro. Although the MIC values for Cro was reduced, the interaction of the two agents was obtained in the “indifferent” category of FICI (FICI: 0.5-4). Therefore, no additive effect was observed when using fluconazole. Moreover, the MIC values were similar for both fluconazole -susceptible and -resistant isolates. 

Consequently, the mechanism of action of Cro is not related to 14-a-demethylase enzyme, which is the target for azole drugs. According to the literature, synergistic effects mostly depend on the concentration of the compounds [27]. In order to determine whether Cro has potent synergistic effect with fluconazole, it is required to select other concentrations, which significantly reduce the MIC of fluconazole against different species of *Candida* by checkerboard microdilution assay. Unlike the current study, the previous studies that used the petroleum ether and methanolic extracts of saffron flower demonstrated that these compounds showed a strong activity against bacteria and fungi. 

The high MIC value for Cro against *Candida* species indicated that this agent failed to show any appropriate antifungal activities. The MIC of this agent was not significantly reduced even by the addition of fluconazole. Therefore, other mechanisms, which are not related to the mechanism of azole drugs, are involved at high concentration of Cro.
